# Role of Age and Education as the Determinant of Income Inequality in Poland: Decomposition of the Mean Logarithmic Deviation

**DOI:** 10.3390/e24060773

**Published:** 2022-05-30

**Authors:** Ewa Wędrowska, Joanna Muszyńska

**Affiliations:** 1 Department of Economic Applications of Informatics and Mathematics, Faculty of Economic Sciences and Management, Nicolaus Copernicus University in Toruń, ul. Gagarina 13a, 87-100 Toruń, Poland; 2 Econometrics and Statistics Department, Faculty of Economic Sciences and Management, Nicolaus Copernicus University in Toruń, ul. Gagarina 13a, 87-100 Toruń, Poland; jmus@umk.pl

**Keywords:** Mean Logarithmic Deviation, Shannon entropy, income inequality, household income, decomposition of income inequality, EU-SILC

## Abstract

Measures of inequality can be used to illustrate inequality between and within groups, but the choice of the appropriate measure can have different implications. This study focused on the Mean Logarithmic Deviation, the measure proposed by Theil and based on the techniques of statistical information theory. The MLD was selected because of its attractive properties: fulfillment of the principle of monotonicity and the possibility of additive decomposition. The following study objectives were formulated: (1) to assess the degree of inequality in the population and in the distinguished subgroups, (2) to determine the extent to which education and age influence the level of inequality, and (3) to ascertain what factors contribute to changes in the level of inequality in Poland. The study confirmed an association between the level of education and the average income of the groups distinguished on this basis. The education level of the household head remains an important determinant of household income inequality in Poland, despite the decline in the “educational bonus”. The study also found that differences in the age of the household head had a smaller effect on income inequality than the level of education. However, it can be concluded that the higher share of older people may contribute to an increase in income inequality between groups, as the income from pension in Poland is more homogeneous than the income from work in younger groups. Moreover, the current paper seeks to situate Theil’s approach in the context of scholarly writings since 1967.

## 1. Introduction

It is well-known that entropy can be considered a measure of uncertainty in probability distribution [1]. Historically, numerous definitions of entropy have been advanced [2,3,4,5,6,7,8] that subsequently are encountered in numerous contexts (thermodynamics, statistical mechanics, information theory, topological dynamics, economy, etc.). The most broadly recognized is Shannon’s information entropy [5]. Statistical entropy introduced by Shannon is an essential concept in information theory, quantifies the unevenness of the probability distribution [9], and may be interpreted as an index of concentration [10]. Furthermore, it needs the straightforward condition of being additive, which was adopted as one of the functional requirements of entropy. According to Theil [11], these properties made it legitimate for entropy to be employed to devise measures that served to quantify income inequality, while the indexes the latter introduced, i.e., the Theil index (TI) and the Mean Logarithmic Deviation (MLD), entered widespread application as instruments for computing concentration and inequality of income distribution. Then, by subtracting Shannon’s entropy of the actual distribution of income shares from the maximum possible value of this entropy, one arrives at the Theil index. Both the TI and the MLD are instances of application of relative entropy or Kullback–Leibler divergence between two income distributions, i.e., actual distribution and equal distribution. Simultaneously, relative entropy introduces alternative variants of the comparative distribution for each Theil measure [12].

Having advanced income inequality measures based on the techniques of statistical information theory, Theil put forward another intellectual concept to define and measure inequality. Not only did he pioneer using entropy to determine inequality, but he also contributed an important set of functional forms by which inequality could be modeled and analyzed. Thanks to Theil, the concepts of information theory became the cornerstone of a new understanding of inequality in income distribution, where it was construed as a discrepancy between the actual income distribution and an implicitly egalitarian reference distribution or some other distribution of economic relevance. In a sense, Theil focused on inequality as a “by-product” of the information content in the income distribution structure. Cowell [13] goes as far as stating that this landmark in comprehending inequality may not have been fully appreciated for some time, and Theil’s contribution may have been more far-reaching than usually assumed. Indeed, there is an extensive amount of works in the literature offering cogent arguments favoring the information theory techniques Theil innovatively employed in studying income inequality. Theil’s proposal engendered a discussion on various theoretical aspects resulting from using information theory methods in the study of inequality. First, one could cite a more flexible general class of measures introduced by Cowell [12] and Cowell and Kuga [14] known as the Generalized Entropy (GE) measures, in which the two Theil indices are special instances. The GE measures constitute the single-parameter entropy family, and their concept is based on comparing observed income distribution with a reference distribution using Csiszár’s divergence (f-divergence) [15]. Talih [16] observes that the GE class is a special case of alpha divergence. The idea of applying dissimilarity measures deriving from the information theory in the study of income inequality was elaborated by References [17,18,19,20,21,22,23]. Second, numerous papers have been devoted to the axiomatization of Theil indices [14,24,25] and, in particular, to the capacity of GE measures (including the TI and the MLD) to additively decompose into within-group and between-group inequalities [17,26,27,28]. Third, the innovative methods introduced by Theil led to a debate on measures of inequality derived from information theory from “ethical” (or prescriptive), as well as “statistical” (or descriptive) standpoints [13,29].

One common application of Theil indices involves the relation between inequality of total income and the values obtained for population subgroups. Theil indices can be easily resolved into terms interpreted as measures of within-group and between-group inequality for population subgroups. This property of additive decomposition is an attractive feature of both Theil indices, promoting numerous applications of the TI and the MLD in the study of the degree of inequality and when examining the factors behind it [27,30,31,32,33,34].

The total income inequality of a population is generated within groups (sub-populations) and between groups. Following this premise, measures based on entropy—which can be easily aggregated (and disaggregated)—are extremely useful for analyzing income inequality in a population divided according to identifiable sociodemographic characteristics such as place of residence, race, gender, age, education, labor market status, etc. The decomposition of an inequality measure enables one to determine how much of the total income inequality can be attributed to variation within groups or, alternatively, to differences between groups. The emergence of comprehensive and detailed micro-data sets made it possible to examine the extent of inequality between people or households. In this study, attention was focused on the equivalized income among Poles in 2005 and 2019. Using EU-SILC data, the age and education of the household head were considered as potential factors of income inequality. Taking these circumstances into account, the following study objectives were formulated: (1) to assess the degree of inequality in the population and in the distinguished subgroups, (2) to determine the extent to which education and age influence the level of inequality, and (3) to ascertain what factors (changes in population structure and changes in income distribution and/or income inequality within subgroups) contribute to changes in the level of inequality in Poland. Therefore, the following hypotheses were formulated:

1. Changes in the age structure of the population in Poland (population aging) increase the importance of age as a determinant of income inequality.
2. Due to the increase of well-educated persons in the share of population, the effect of differences in the level of education on the level of income inequality decreases.

Moreover, the current paper seeks to situate Theil’s approach in the context of scholarly writings since 1967. Specifically, the Mean Logarithmic Deviation is considerably highlighted, given that the Theil index treats differences in all parts of the distribution equally, while the MLD is more responsive to changes at the bottom tail [35]. The Mean Logarithmic Deviation was selected from among all additively decomposable inequality measures because it is the sole measure that allows unequivocal partitioning of the total inequality due to differences between subgroups [27]. The decomposition methodology relies on an a priori approach which derives from theoretical axioms and employs a decomposition technique by population subgroups consistent with Shorrocks [17,27] and Mookherjee and Shorrocks [36].

The structure of this paper is as follows: Section 2 outlines the conceptual background, particularly the intellectual basis for defining the MLD, along with its properties and decomposition techniques based on the axiomatic approach. Section 3 describes the dataset used in the study. The subsequent section provides the results of the empirical analysis, while the conclusions are stated in the final section.

## 2. Conceptual Background

### 2.1. Notation and Concepts

To clarify the notation used throughout the paper, let $y_{i}\in R_{+}$ represent the (positive) individual income of a person *i* (i=1,2,…, n), and $y:=\left(y_{1},y_{2},\ldots ,y_{n}\right)\in R_{+}^{n}$ is the income distribution vector for a population of *n* individuals. The set of all possible income distributions, *D*, was also defined with specific income distribution, $y\in R_{+}^{n}$, and mean income level, $\mu =\mu \left(y\right)=\frac{Y}{n}$, where $Y=\sum_{i=1}^{n}y_{i}$ denotes the total number of income units. A parameter vector θ(y)=(μ(y),n(y)) of the distribution *y* is introduced following Shorrocks [17], where n(y)=n denotes the dimension of any vector *y* (i.e., the population size).

Mean income is most often adopted as a natural point of reference in the study of income inequality [37]. For this reason, vector $\bar{y}:=\left(\mu ,\mu ,\ldots ,\mu \right)\in R_{+}^{n}$ will be employed to signify the equalized version of *y*. Vector $\bar{y}$ represents a perfectly equal distribution in a situation in which the income of all persons is equal. The mean vector, $\bar{y}$, shall be referred to as the reference distribution for perfect equality.

The connection between information theory and the economic interpretation of income distributions is established by exploiting the close relationship between entropy measures (based on probability distributions) and measures of inequality (based on distributions of income shares) (Reference [22], p. 422). Therefore, based on individual income for each *i* person, it is possible to determine their share in total income, Y: $q_{i}=\frac{y_{i}}{Y}$. The actual vector of income shares is structured as $q:=\left(\frac{y_{1}}{Y},\frac{y_{2}}{Y},\ldots ,\frac{y_{n}}{Y}\right)=\left(\frac{y_{1}}{n\mu },\frac{y_{2}}{n\mu },\ldots ,\frac{y_{n}}{n\mu }\right)$, whereas the vector of equal income shares is as follows: $p:=\left(\frac{1}{n},\frac{1}{n},\ldots ,\frac{1}{n}\right)$.

Finally, the designated notation will be used to describe the division of *n* income-receiving units into G≥2 mutually exclusive and exhaustive subgroups (e.g., by education, age, gender, race, occupation, or region). The overall distribution, *y*, is partitioned into *G* subgroup distributions: $y=\left(y^{1},y^{2},\ldots ,y^{G}\right)$. The mean income of the *g* subgroup is denoted as $\mu^{g}=\mu \left(y^{g}\right)$, while $n^{g}=n\left(y^{g}\right)$ describes its numerical strength ($\sum_{g=1}^{G}n^{g}=n$). Let $\bar{\mu }=\left(\mu^{1},\mu^{2},\ldots ,\mu^{G}\right)$, and $\bar{n}=\left(n^{1},n^{2},\ldots ,n^{G}\right)$ represents the vectors of subgroup means and population sizes, respectively. For each distribution *y^g^* (g=1,…,G), the parameter vector is as follows: $\theta^{g}\left(y^{g}\right)=\left(\mu \left(y^{g}\right),n\left(y^{g}\right)\right)=\left(\mu^{g},n^{g}\right)$. Furthermore, $v^{g}=\frac{n^{g}}{n}$ denotes subgroup population shares, $\lambda^{g}=\frac{\mu^{g}}{\mu }$ stands for relative mean income, and $\theta^{g}=v^{g}\lambda^{g}$ signifies *g* group income share in the income of the entire population.

An inequality measure is normally a real-valued function, I(y):D→R, which is given meaning by axioms that integrate criteria derived from ethics, intuition, or mathematical convenience [13]. In the pertinent literature, the Mean Logarithmic Deviation index is most often presented by using the formula below:

(1) $$MLD=\frac{1}{n}\sum_{i=1}^{n}\ln\frac{\mu }{y_{i}}=-\frac{1}{n}\sum_{i=1}^{n}\ln\frac{y_{i}}{\mu }.$$

However, for a comprehensive picture of inequality in income distribution as a concept of disparity between the actual income distribution, *y*, and the equal distribution, $\bar{y}$, based on information theory techniques, Formula (1) is expressed as follows:

(2) $$MLD=\frac{1}{n}\sum_{i=1}^{n}\left[\ln\mu -\ln y_{i}\right]$$

or

(3) $$MLD=\frac{1}{n}\sum_{i=1}^{n}\ln\frac{\mu }{y_{i}}=\frac{1}{n}\sum_{i=1}^{n}\ln\frac{\frac{y_{i}}{nq_{i}}}{y_{i}}=\frac{1}{n}\sum_{i=1}^{n}\ln\frac{1}{nq_{i}}=\frac{1}{n}\sum_{i=1}^{n}\left[\ln\frac{1}{n}-\ln q_{i}\right].$$

Formula (2) represents the average deviation between average income logarithm, *μ*, and income logarithm, $y_{i}$. In turn, Formula (3) analogously allows for the combined concept of distance between vectors *q* and *p*. It may thus be said that it represents the average deviation between the income share logarithm and the logarithm of shares which would constitute perfect equality.

Straightforward conversions of the Formula (2) make it possible to demonstrate that the *MLD* index is the difference between the logarithm of the arithmetic mean of income (*μ*) and the logarithm of the geometrical mean (*μ_g_*):

(4) $$MLD=\frac{1}{n}\sum_{i=1}^{n}\left[\ln\mu -\ln y_{i}\right]=\frac{1}{n}\sum_{i=1}^{n}\ln\mu -\frac{1}{n}\sum_{i=1}^{n}\ln y_{i}=\ln\mu -\frac{1}{n}\ln\left(\prod_{i=1}^{n}y_{i}\right)=\ln\mu -\ln\mu_{g}.$$

Ultimately, it may be shown that the Mean Logarithmic Deviation is simply tantamount to the Kullback–Leilber divergence (KL divergence), in which prior distribution constitutes the actual vector of income shares, $q=\left(\frac{y_{1}}{n\mu },\frac{y_{2}}{n\mu },\ldots ,\frac{y_{n}}{n\mu }\right)$, whereas posterior distribution is the vector of equal income shares, $p:=\left(\frac{1}{n},\frac{1}{n},\ldots ,\frac{1}{n}\right)$:

(5) $$D\left(p\|q\right)=\sum_{i=1}^{n}p_{i}\ln\left(\frac{p_{i}}{q_{i}}\right)=\sum_{i=1}^{n}\frac{1}{n}\ln\left(\frac{\frac{1}{n}}{\frac{y_{i}}{n\mu }}\right)=\sum_{i=1}^{n}\frac{1}{n}\ln\frac{\mu }{y_{i}}=MLD.$$

D(p‖q) measures the divergence between actual income distribution, *q*, and the perfectly equal distribution, *p*. Taking advantage of the interpretation known in information theory, it may be said that D(p‖q) quantifies the amount of information obtained following a transformation of the prior distribution (*q*) into posterior distribution (*p*). D(p‖q) is a measure of the information lost when *q* is used to approximate *p*. The Kullback–Leilber divergence may also be construed as a measure of surprise [38]. Therefore, the MLD is the surprise in transitioning from the income distribution one actually has to a hypothetical equal distribution. As is commonly known, the KL divergence is non-symmetric: (D(p‖q)≠D(q‖p)) and D(p‖q)≥0, whereby its value is equal to 0 when the compared distributions are identical. When quantifying the level of income inequality, if it is assumed that the reference distribution is even, it is evident that D(p‖q)=0 when the examined distribution is $q=\left(\frac{1}{n},\frac{1}{n},\ldots ,\frac{1}{n}\right)$. Consequently, the *MLD* index assumes 0 value if (and only if) income distribution, *y*, is completely equal, meaning that the MLD index meets the property known as normalization.

The Kullback–Leibler divergence does not have an upper-bound. The maximum value of the *MLD* depends on how small an income is determined for *y_i_*. This may be a certain shortcoming of the *MLD* index in the assessment of income inequality, in particular when contrasted with the Gini coefficient, for example, which also easily yields to interpretation. Nonetheless, the many attractive properties of the *MLD* that come to light in the next section have made it one of the commonly used measures of inequality.

The Mean Logarithmic Deviation belongs to a more flexible general family of inequality measures. Cowell extended the class of inequality measures based on information theory techniques to the Generalized Entropy measures [12,14]:

(6) $$GE_{\alpha }\left(y\right)=\{\begin{array}{cc} \sum_{i=1}^{n}\left({\left(y_{i}/\mu \right)}^{\alpha }\right)/n\left(\alpha^{2}-\alpha \right) & \alpha \neq 0,1\\ -\sum_{i=1}^{n}\ln\left(y_{i}/\mu \right)/n & \alpha =0\\ \sum_{i=1}^{n}\left(y_{i}/\mu \right)\ln\left(y_{i}/\mu \right)/n & \alpha =1 \end{array},$$

where αϵ(−∞,+∞) is a parameter capturing the sensitivity of a particular GE measure to different parts of the distribution. This class includes, as is widely acknowledged, the Theil index (for α=1), the Mean Logarithmic Deviation (for α=0), and monotonic transformations of the coefficient of variation of the entire Atkinson family of indices. The GE measures constitute a class of relative indices which are normalized by the mean.

The Generalized Entropy measures offer the ability to examine the effects of inequalities in different areas of the income spectrum, enabling more meaningful quantitative assessments of qualitatively different inequalities. As α decreases, the measure’s sensitivity to the lower tail, i.e., the poor, increases. Cowell [13] observes that GE is more sensitive to income variation in the upper tail of distribution for high and positive α, whereas, with negative α, the index becomes sensitive to income variation in the lower tail of distribution. In particular, the MLD displays greater sensitivity to changes at the bottom tail.

### 2.2. Axiomatic Approach

Comparative reference, which provides the basis for inequality measurement using techniques of the statistical information theory, is usually a permanent component of investigations into inequality. Often enough, it is not transparent without verifying mathematical properties which lie at the foundation of the structure of a given inequality measure. Roberto [39] argues that there are many measures which operationalize any given dimension of inequality and divides inequality measures with respect to two dimensions: evenness and diversity. Coulter [40], on the other hand, split inequality measures into four categories in accordance with their basic mathematical model: combinatorics, entropy, deviations, and social-welfare function. However, the most widely adopted classification criterion is the link between measures of inequality and the concept of social welfare. According to Sen [41], measures of inequality can be divided into two broad classes: normative and positive. The former measure the level of inequality in terms of the normative notion of social welfare and the loss incurred as a result of unequal income distribution. They rely on the “ethical” link to social-welfare functions developed by Kolm [42], Atkinson [43], and Sen [41] and are thus informed by value judgements. Conversely, positive measures do not make explicit use of the concept of social welfare and serve only a descriptive function, suitably conveying the degree of inequality in an appropriate way and help one to assess the significance of the impact of various factors. However, Sen [41] notes that this division is not precise since any positive measure of inequality is always entangled with a social welfare function. He cites the Theil index, which, in terms of form, almost fully corresponds with the utilitarian social-welfare function, as a result of which individual welfare components are equal to $y_{i}\ln\left(\frac{1}{y_{i}}\right)$ (see Reference [41], p. 43). Shorrocks [29] adds that the descriptive and ethical aspects of inequality measures are complementary.

In describing the properties of the *MLD* index, an axiomatic approach will be used which seeks to characterize measures that satisfy relevant properties in the context of income inequality analysis. Magdalou [44] underlines that such an approach usually yields a unique class of indices which the Generalized Entropy measures represent.

The *MLD* index satisfies five key axioms: symmetry, the principle of population replication, scale invariance, the Pigou–Dalton transfer principle, and an additive decomposition property. It may be interesting to note that any inequality measure that satisfies these axioms must belong to the Generalized Entropy class or its ordinal transformations [27].

1. *Symmetry*. Let $y^{\prime }=\left({y^{\prime }}_{1},\;{y^{\prime }}_{2},\;\ldots ,{y^{\prime }}_{n}\right)\in D$, which is obtained from $y=\left(y_{1},\;y_{2},\;\ldots ,y_{n}\right)\in D$ by a permutation of incomes $y_{i}$.
  $MLD\left(y^{\prime }\right)=MLD\left(y\right)$ whenever *y*′ is obtained from *y* by a permutation.
2. *Principle of Population Replication (Replication Invariance)*. Let x=(y, y, …, y)∈D, which is obtained from $y=\left(y_{1},\;y_{2},\;\ldots ,y_{n}\right)\in D$ by a replication. The incomes in *x* are simply the incomes in *y* repeated a finite number of times. If *x* is obtained from *y* by a replication:
  MLD(x)=MLD(y) whenever *x* is obtained from *y* by a replication.
3. *Scale Invariance (Scale Independence)*. Let $x=\left(\alpha y\right)=\left(\alpha y_{1},\alpha y_{2},\ldots ,\alpha y_{n}\right)\in D$, which is obtained from $y=\left(y_{1},\;y_{2},\;\ldots ,y_{n}\right)\in D$ by a scalar multiple for some positive real α.
  MLD(x)=MLD(y) whenever *x* is obtained from *y* by a scalar multiple.
4. *The Pigou–Dalton Transfer Principle*. Let $x=\left(x_{1},\;x_{2},\;\ldots ,y_{i}-t,\ldots ,y_{j}+t,\ldots ,x_{n}\right)\in D$, which is obtained from $y=\left(y_{1},\;y_{2},\;\ldots ,y_{i},\ldots ,y_{j},\ldots ,y_{n}\right)\in D$ by regressive transfer t>0. This means that, for any given income value, *y_i_* and *y_j_*, which satisfy $y_{i}<y_{j}$, transfer t>0 proceeds as follows: $y_{i}-t=x_{i}$ and $y_{j}+t=x_{j}$, $x_{i}\leq x_{j}$, whereas, for any given k≠i,j, we obtain $x_{k}=y_{k}$ ($y_{i}-x_{i}=x_{j}-y_{j}>0$).
  MLD(x)=MLD(y) whenever *x* is obtained from *y* by a regressive transfer.
5. *Additively decomposable*. Suppose that the overall distribution *y* is partitioned into *G* subgroup distributions: $y=\left(y^{1},y^{2},\ldots ,y^{G}\right)$. Additive decomposition property is defined according to References [20,27,45]. The decomposition formula will make use of a parameter vector $\bar{\theta }=\left(\bar{\mu },\bar{n}\right)\in R_{+}^{G}\times N^{G}$ for both the weights employed in the within-group term and the distribution used to define the between-group term. For each vector $\bar{\theta }=\left(\bar{\mu },\bar{n}\right)\in R_{+}^{G}\times N^{G}$, let us define a weighting function, $w\left(\bar{\mu },\bar{n}\right):=\left(w^{1}\left(\bar{\mu },\bar{n}\right),w^{2}\left(\bar{\mu },\bar{n}\right),\ldots ,w^{G}\left(\bar{\mu },\bar{n}\right)\right)$, for which $w^{g}\left(\bar{\mu },\bar{n}\right)\geq 0$ is the weight attached to the *g* subgroup inequality, assuming that it depends on $\bar{\mu }$ and $\bar{n}$. The between-group term is based on the smoothed distribution, $\chi \left(\bar{\mu },\bar{n}\right):=\left(\mu^{1}1^{n^{1}},\mu^{2}1^{n^{2}},\ldots ,\mu^{G}1^{n^{G}}\right)$, which replaces the income of each person in the *g* subgroup with a correspondingly mean subgroup income, $\mu^{g}$ ($1^{n^{g}}$ is *n^g^*-coordinated vector of ones).

The *MLD* is additively decomposable, as shown below:

(7) $$MLD\left(y^{1},y^{2},\ldots ,y^{G}\right)=\sum_{g=1}^{G}w^{g}\left(\bar{\mu },\bar{n}\right)MLD\left(y^{g}\right)+MLD\left(\chi \left(\bar{\mu },\bar{n}\right)\right).$$

Belonging to the family of Generalized Entropy measures, the *MLD* satisfies the above measure properties for that class. Still, the *MLD* is a particular instance of median-normalized inequality measures suggested by Reference [37]. These measures were devised by using the median (*m*) as the equality reference point; in contrast, mean income is adopted as a reference point for the mean-normalized GE.

A median-based class of Generalized Entropy inequality measures is defined as follows:

(8) $$I_{\alpha }\left(y/m;\mu \right):=\frac{1}{\alpha \left(\alpha -1\right)}\frac{1}{n}\sum_{i=1}^{n}\left[{\left(\frac{y_{i}}{m}\right)}^{\alpha }-{\left(\frac{\mu }{m}\right)}^{\alpha }\right],\;\alpha \neq 0,1.$$

By applying de l’Hôpital’s rule for α=0 and α=1, we obtain the following, respectively:

(9) $$I_{0}\left(y/m;\mu \right):=-\frac{1}{n}\sum_{i=1}^{n}\ln\frac{y_{i}}{\mu },$$

(10) $$I_{1}\left(y/m;\mu \right):=\frac{1}{n}\sum_{i=1}^{n}\frac{y_{i}}{m}\ln\frac{y_{i}}{\mu }.$$

Let us note that the particular case of the median-normalized inequality measures for α=0 is independent of the median, and Formula (9) describes the Mean Logarithmic Deviation index.

Cowell and Flachaire [37] demonstrated that the median-normalized inequality measures satisfy the property known as monotonicity in distance for α≥0. The principle of monotonicity in distance was advanced by Cowell [18] as a certain generalization of the transfer principle. Magdalou and Nock [21] draw attention to the fact that the property is “quite demanding”. The principle of monotonicity in distance means that a departure of any income, *y_i_*, from the reference point, *e*, should be interpreted as an increase of inequality.

### 2.3. The MLD Decomposition by Subgroups

The decomposition criterion is to be met by inequality measures when the assessment of the level of income inequality in a population involves the premise that inequality is not an inherent characteristic of the community but a certain function of its component elements. It is assumed that the level of inequality in the entire society is contingent on the degree of inequality in the subgroups isolated on the basis of certain traits, and precisely those traits. This study sought to establish how the overall level of inequality may be decomposed into contributions resulting from (1) income inequality within each subgroup and (2) inequality between groups that arise from differences between the mean level of income in those subgroups.

Shorrocks [27] identified a class of relative inequality measures which are additively decomposable by subgroups. The Generalized Entropy measures—inclusive of the MLD—constitute a family of those measures. Subgroup decomposable indices are also known in the literature as additively decomposable or, more concisely, additive indices [45].

Let us assume yet again that the studied population into *G* of mutually exclusive and exhaustive subgroups. The Mean Logarithmic Deviation is additively decomposable in accordance with Formula (7), in which weights $w^{g}\left(\bar{\mu },\bar{n}\right)$ linked to the *g* subgroup income level equal $v^{g}=\frac{n^{g}}{n}$. In other words, the within-group term $MLD_{W}$ is a weighted sum of inequalities in subgroups, and it is expressed in the following formula:

(11) $$MLD_{W}=\sum_{g=1}^{G}w^{g}\left(\bar{\mu },\bar{n}\right)MLD\left(y^{g}\right)=\sum_{g=1}^{G}\frac{n_{g}}{n}MLD\left(y^{g}\right).$$

The within-group component describes a part of the overall inequality which is due to the inequalities in the subgroups.

Between-group term $MLD_{B}$ quantifies income inequalities in the smoothed distribution $\left(\mu^{1}1^{n^{1}},\mu^{2}1^{n^{2}},\ldots ,\mu^{G}1^{n^{G}}\right)$, which would result from replacing each income, *y_i_*, with the *g* subgroup mean income, in which the *i* person is classified. Therefore, the between-group component $MLD(\chi \left(\bar{\mu },\bar{n}\right)$ found in Formula (7) may be expressed as follows:

(12) $$MLD_{B}=\sum_{g=1}^{G}\frac{n_{g}}{n}\ln\left(\frac{\mu }{\mu_{g}}\right).$$

In other words, the between-group component describes the scale of inequality resulting exclusively from the differences in mean income of the subgroups. The ratio of inequality between subgroups to overall inequality reflects the extent to which the feature on the basis of which the subgroups are distinguished contributes to household income inequality [46].

Theil [11] formulated a vital requirement that subgroup decomposable inequality measures should satisfy. Specifically, the within-group and their associated weights should be independent of the between-group component. This is because a major problem arises when mutually independent within-group and between-group terms cannot be defined. Shorrocks [27] and Anand [47] also highlight the problem, stressing that changes in inequality between groups can induce modifications not only in the between-group component but also in the within-group component, even if within-group income inequality has registered no change. With the issue in mind, Foster and Shneyerov [48] examine an additive decomposition property which they refer to as “path independent decomposability”. This property requires that the between-group and within-group components be independent. Foster and Shneyerov [48] introduced a class of inequality measures which satisfy this property, designated as path-independent indices. Path-independent inequality measures have within-group terms that can be expressed as population-share weighted sums of the subgroup inequalities. Path-independent inequality measures have within-group terms that can be expressed as population-share weighted sums of the subgroup inequalities. The researchers demonstrate that the Mean Logarithmic Deviation and the variance of logarithms $V_{L}=\sum_{i=1}^{n}{\left(\ln y_{i}-\ln\mu_{g}\right)}^{2}/n$ belong to this class and, thus, satisfy the path-independent decomposability property. The *MLD* index has a path-independent decomposition that uses the arithmetic mean as the representative income, while the variance of logarithms is path-independent relative to the geometric mean.

Another relevant issue involved in the interpretation of the results obtained from the decomposition of inequality measures was identified by Shorrocks [27]. This problem concerns the interpretation of statements such as “X per cent of inequality is due to Y”. To understand this issue better, let us consider a possible answer to the question “What proportion of total inequality is due to income differences due to attribute (characteristic) Y?” This may be interpreted as follows: (1) What decrease in inequality will be observed if differences in the studied attribute Y are the only source of income differences? (2) How far will inequality decrease if income differences due to attribute Y disappear? Since these interpretations are equivalent modes of answering the same question, the inequality measure is expected to yield the same answer for both cases (1) and (2). Only inequality measures for which the weights of the within-group expressions do not depend on the subgroup means will generate the same answer for (1) and (2). Given that GEs are additively decomposable with weights $w^{g}\left(\bar{\mu },\bar{n}\right)=\left(\frac{n^{g}}{n}\right){\left(\frac{\mu^{g}}{\mu }\right)}^{\alpha }$ [20,27], there is only one member of the Generalized Entropy family to satisfy this property, namely the *MLD*. Therefore, Shorrocks (Reference [27], p. 625) underlines that the *MLD* “is the most satisfactory of the decomposable measures, allowing total inequality to be unambiguously split into the contribution due to differences between subgroups”.

The decomposition of inequality indices by population subgroups has often been used to explain trends in income distribution. With the population divided into subgroups, the decomposed inequality (into within- and between-group components) in a given year may be expressed as a function of three components: subgroup population shares, subgroup mean incomes, and subgroup inequalities. The change in inequality between the two examined periods can therefore be linked to changes in these three components. Therefore, three main components are identified in the decomposition of the inequality trend, corresponding respectively to the following: changes in population structure ($\Delta v^{g})$, relative fluctuations in subgroup mean incomes ($\Delta \ln\mu_{g}$), and changes in subgroup inequality values ($\Delta MLD\left(y^{g}\right))$ [36]. Since the *MLD* is an index for which indices expressing subgroup inequality are weighted by subgroup shares in a population, Mookherjee and Shorrocks [36] show that changes in inequality between two periods (*t*_0_ and *t*_1_) can be noted as follows:

(13) $$\Delta MLD=MLD\left(t_{1}\right)-MLD\left(t_{0}\right)\;=\sum_{g=1}^{G}{\bar{v}}^{g}\Delta MLD\left(y^{g}\right)+\sum_{g=1}^{G}\bar{MLD}\left(y^{g}\right)\Delta v^{g}-\sum_{g=1}^{G}\bar{\ln\lambda^{g}}\Delta v^{g}-\sum_{g=1}^{G}{\bar{v}}^{g}\Delta \ln\lambda^{g},$$

where Δ is the difference operator, and a bar over variables indicates an average of base and current period value ($v^{g}=\left(v^{g}\left(t_{0}\right)+v^{g}\left(t_{1}\right)\right)/2$, etc.).

The MLD can be approximately decomposed into the contributions of changes in inequality within groups, changes in inequality between groups, changes in the population share of each group, and changes in the subgroup means:

(14) $$\begin{array}{cc} \begin{array}{cc} \approx \sum_{g=1}^{G}{\bar{v}}^{g}\Delta MLD\left(y^{g}\right) & +\sum_{g=1}^{G}\bar{MLD}\left(y^{g}\right)\Delta v^{g}\\ \left[\mathrm{term}A\right] & \left[\mathrm{term}B\right] \end{array} & \begin{array}{cc} +\sum_{g=1}^{G}\left({\bar{\lambda }}^{g}-\bar{\ln\lambda^{g}}\right)\Delta v^{g} & +\sum_{g=1}^{G}\left({\bar{\theta }}^{g}-{\bar{v}}^{g}\right)\Delta \ln\mu_{g}\\ \left[\mathrm{term}C\right] & \left[\mathrm{term}D\right] \end{array} \end{array},$$

The term A of the expression (14) represents the impact of changes in within-subgroup inequality (“pure” inequality changes); terms B and C indicate the effect of changes in the population shares on the within-group and between-group components (allocation effect); and term D (income effect) is the contribution to overall inequality change attributable to relative changes in the subgroup means [34,36].

Formula (14) is employed to assess changes in inequality, because, as Jenkins [34] argues, “the approximation is more useful than the exact decomposition because it relates inequality changes to changes in subgroup inequalities, shares and means (rather than relative means)”.

## 3. Data

The current study employed data from the EU-SILC survey conducted by Eurostat. The EU-SILC is an annual EU-wide household survey which provides information on the income and living conditions of a sample of households. The micro-data were obtained under the project entitled *Income and inequality of income of European households* (RPP 162/2018-EU-SILC). The current study used data extracted from the cross-sectional database of the EU-SILC 2019 (version as of March 2021). As the EU-SILC adopts the preceding year as the income reference period for the vast majority of countries, including Poland, the data taken from 2005 and 2019 surveys cover information about incomes achieved by individual members of Polish households in 2004 and 2018, respectively.

Considering data availability, in 2005 and 2019, the samples of raw micro-data obtained from the cross-sectional EU-SILC data set spanned 16,263 households, i.e., 49,044 individuals and 19,874 households with 50,788 persons, respectively. Due to the lack of information on the education and age of the household head, the number of observations was limited to 48,916 individuals in 2005 and 43,935 persons in 2019. In the analysis, the authors also omitted 117 observations with zero and negative incomes in 2005 and disregarded 103 such observations in 2019. All the inequality measures reported in the study were calculated by using personal cross-sectional weights.

Throughout the empirical analysis, the authors employed the annual equivalized household disposable income per household member. The total household disposable income was calculated as a sum of gross personal income components for all household members and gross income components at the household level reduced by taxes, social insurance contributions, and inter-household cash transfers paid. To compensate for different household structures and possible economies of scale within households, household income was size-adjusted by dividing total income by the equivalized household size and assigning this value to each household member. To size-adjust household disposable income, the study used the OECD-modified equivalence scale, which assigns a weight of 1.0 to the head of household, 0.5 to every household member aged 14 or more, and 0.3 to each child aged under 14. Summing up the individual weights gives the equivalized household size.

For years, income inequality (as well as its sources) has been the subject of numerous studies. The micro-determinants of household income inequality, referred to in the relevant literature, can be assembled into two groups: sources of household income and sociodemographic attributes associated with the household and its members. In the current study, we focused on the personal characteristics of household members, specifically the head of household, and considered two possible drivers of inequality: the level of education and the age of the household head.

Various approaches to selecting the head of the household can be found in empirical research. Very often, the household head was considered to be the member of the household who had made the largest contribution to the household income [33,49]. Kranziger [50] designated the oldest member of the household as its head, while, in Papatheodorou’s analysis [51] of inequality in Greece, the male was presumed to be the head of the household. In the current study, Medgyesi’s approach [52] was employed; it defined the household head on a demographic basis. The oldest male of working age (16–64 years old) in the household was considered to be the head of the household. In case there was no such individual, the oldest working-age female was taken as the household head. If no working-age persons resided in the household, the oldest male member was considered the household head, or, alternatively, the oldest female was. We decided to follow Medgyesi because, due to the economic situation in Poland, housing shortages, as well as Polish culture, many young, well-educated people with high incomes share dwellings with their relatives. Even if their income exceeds that of their parents, the oldest working male (usually the father) is still considered the head of the household.

**Figure 1:**
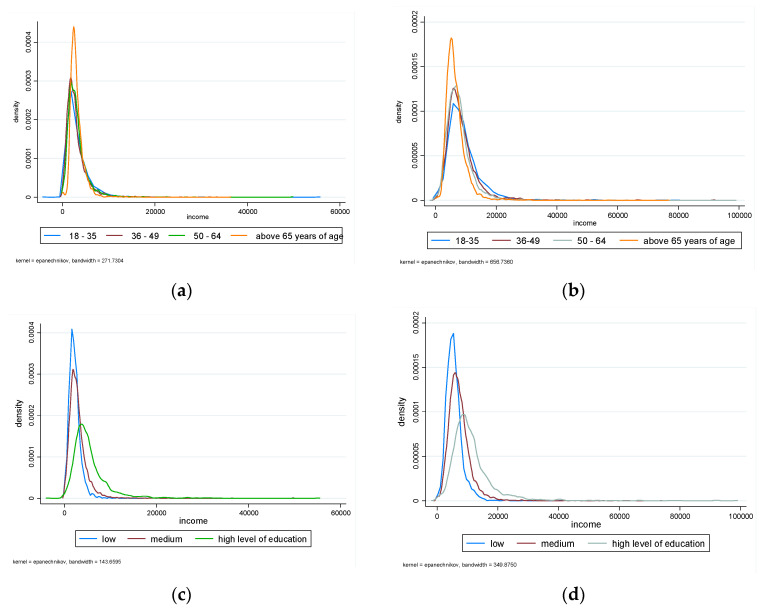
The Kernel density estimates of equivalized disposable income for the subgroups were distinguished based on the age of the household head in 2005 (**a**) and 2019 (**b**), and for the subgroups distinguished by the education of the household head in 2005 (**c**) and 2019 (**d**), respectively. Source: Own elaboration based on the EU-SILC data.

For the age of the head of household, the following household subgroups were distinguished: 18–35, 36–49, 50–64, and above 65 years of age. Figure 1a,b presents the estimation of the Kernel density of equivalized disposable income for the subgroups, which were distinguished based on the age of the household head in 2005 (Figure 1a) and 2019 (Figure 1b), respectively.

The estimation of the Kernel density of equivalized disposable income for the subgroups distinguished by the education of the household head in 2005 (Figure 1c) and 2019 (Figure 1d), respectively, is presented in Figure 1c,d.

In the SILC, the level of education is assigned according to the International Standard Classification of Education (ISCED 2011), which comprises nine categories of educational attainment. In the current analysis, it was decided to merge certain education levels and create three subgroups of households with low, medium, and high education levels of the household head. The first subgroup (low level) included early childhood education, primary, and lower secondary education. The subgroup of medium educational level would have completed upper-secondary and post-secondary education. The last subgroup (high level) included short-cycle tertiary, bachelor’s, master’s, doctoral, or equivalent education.

## 4. Empirical Analysis

The first step in the analysis was to estimate the level of household income inequality in Poland in both examined years. The level of income inequality measured by the value of the MLD of equivalized household disposable income amounted to 0.2319 in 2005 and to 0.1465 in 2019. Although the level of income inequality in both years can be considered moderate, there has been a significant decline in inequality between the years in question.

In analyzing income inequality in the current study, the authors focused on the personal attributes of the household head (age and education) as the possible determinant of income disparities between households. An attempt was made to assess the extent to which the selected factors affect household income inequality, as well as whether their impact is stable over time.

Table 1 presents the summary statistics on equivalized disposable income and population of the subgroups of households distinguished relative to the age of the household head.

After dividing the households into four subgroups by the age of the household head, it was found that, in 2005, households headed by a person aged 36–49 were as common as households run by a person aged 50–64. Their share in the population was 37.8% and 36.9%, respectively. In 2005, the smallest share of the population (8.3%) lived in households run by a person aged above 65. While in the first year under study, two subgroups of household accounted for the dominant share of the population, in the second year, there were greater disparities in the proportion of the population. In 2019, the most numerous group of households was headed by a person aged 50–64, comprising over 42% of the population. The share of households run by the oldest persons (above 65) also increased. On the other hand, the shares of households headed by persons aged 36–49 and 18–35 decreased to 34.5% and 12.1%, respectively. The changes observed in the structure of households confirm the aging of Polish society.

An examination of the level and structure of income indicated that, in 2005 the average income of the defined subgroups was very close to the Polish average. The youngest households achieved the highest average income, but it was only 3.8% higher than the national average. The lowest average income was achieved by households run by a person aged 36–49. Their average income was 4.3% lower than the national one. A different situation was observed in 2019, when there was a negative correlation between the age of the household head and the average income in the subgroups. The youngest households achieved the highest average income, exceeding the national by 14.2%. The average income of households headed by a person aged 36–49 was also higher than the national average, but only by 5.4%. The remaining subgroups, run by people aged 50–64 and above 65, achieved lower average income than the national average by 4% and 17.5%, respectively. The unfavorable changes observed in the case of households headed by the oldest persons indicate a deterioration of their relative income situation.

It was also found that, in 2005, the structure of income corresponded to the structure of the population. The largest proportion of income was distributed among the most numerous subgroups, where household heads were aged 36–49 and 50–64, with a slight advantage of the latter. Their shares in the structure of income amounted to 36.1% and 37.8%, respectively. In 2019, households headed by a person aged 50–64 increased their share in the income structure to over 40%, while the share of the youngest households decreased by approximately 4 percentage points. The shares of households run by people aged 36–49 and aged above 65 remained almost unchanged.

The overall income inequality consists of both the differences between average income in the distinguished subgroups and the differentiation of income within these subgroups. Despite the differences in average incomes of the subgroups distinguished by the age of the household head, more inequality was observed within the subgroups than between them. In both examined years, the only subgroup with significantly lower inequality than overall inequality was that of the oldest households. It was also the only subgroup in which there was a slight increase in inequality between the studied years.

With respect to the level of income inequality within the distinguished subgroups, a negative correlation was found between the age of the household head and the level of inequality; that is, the older the head of the household, the lower the level of income inequality within the subgroup. It was also observed that in 2019 a higher average income of the subgroup was accompanied by a higher level of inequality.

To assess whether the age of the household head contributes to household income inequality, we employed the ratio of inequality between subgroups to overall inequality. The results of the *MLD* decomposition are presented in Table 2.

It was determined that the age of the household head was of negligible importance in 2005, and it accounted for 0.3% of overall inequality. However, the importance of this determinant increased significantly in 2019, and the ratio of the between-group component to overall inequality was almost tenfold higher. It may be expected that the observed process of the aging of society will entail a further increase in the importance of the age of the household head as a factor in household income inequality.

The level of education of the household head was the second factor to be analyzed. The summary statistics reflecting equivalized disposable income and population of the subgroups of households distinguished by education of the household head are presented in Table 3. As a result of dividing households on this basis, three subgroups of households were obtained with a “low”, “medium”, or “high” education level of the household head. As regards the structure of the household population, it was found that the subgroup of households run by a person with a medium level of education was the most numerous in both examined years. While the dominant share of households with medium education of the household head was expected, substantial changes were noted in the proportions of the population in the remaining subgroups between the studied years. In 2005, households run by a person with a low level of education accounted for 21.5%, whereas households headed by a person with a high education level constituted 12.7%. In 2019, the opposite was observed. The share of households with a low-educated household head decreased to 11%, while the proportion of households headed by a highly educated person rose to 23%. The observed changes may have been due to “the fashion for having higher education” (even inconsistent with one’s adopted profession) prevailing in Poland in the first decade of the 21st century.

As for the distribution of income among the distinguished subgroups, the share of income of the “medium” group corresponded to its share in the population. Households in this subgroup accounted for 66% of the population and accumulated over 60% of income. The smallest part of income belonged to households headed by a person with a low level of education. In 2005, the share of this subgroup in income accounted for 15.6% and decreased twofold between the studied years, as did its share in the population. A reverse tendency was observed in the subgroup of households run by a person with a high level of education. As in the population, the income share of the “high” subgroup rose by ten percentage points.

The average income of a household run by persons with a low or medium level of education was lower than the average income in Poland and amounted to 70% and 90% of the national average, respectively. The highest average income was observed in the “high” subgroup, although the advantage in relation to the national average decreased by almost half in the examined years, from 77% to 40%. When comparing the average income in the defined subgroups, we found a positive correlation between income and education, and a higher education level of the household head coincided with a higher level of income inequality within the subgroup. In the examined years, the level of within-group inequality decreased significantly. The most substantial drop (over 43%) was recorded in the subgroup of households run by a person with a medium level of education. In the subgroups of households with “low” and “high” education level of the household head, the declines were also considerable, but not that high, and amounted to 36% and 26%, respectively.

The ratio of inequality between subgroups to overall inequality was 16% in both analyzed years (Table 2). This means that, if the average incomes of the subgroups distinguished on the basis of the education of the household head were equal, and within-group inequality remained unchanged, the level of overall income inequality would decrease by 16%. The high ratio confirmed the role of education as a determinant of income inequality. In 2005, the impact of education of the household head on household income inequality was significant, and its importance did not change over time.

In order to identify the factors associated with changes in income inequality in 2005–2019, a dynamic decomposition of the *MLD* was used. The results are presented in Table 4.

The overall change in income inequality was partitioned into four components. The first component, showing the effect of inequality, described the impact of changes on within-group inequality. The allocation effect, broken down into two components, showed how changes in population shares affected the within-group and between-group component, respectively. The latter component represented the income effect. It showed the impact of relative changes in the average incomes of the distinguished subgroups.

In the study, the level of household income inequality was analyzed in two distributions: by age and by the level of education of the household head. As shown in Table 4, the decline in household income inequality in Poland in both distributions resulted chiefly from the decline in within-group inequality. Changes in the population structure by age, i.e., an increase in the share of households run by persons aged 50+, contributed to a decline in the level of income inequality, reducing the within-group component. On the other hand, relative changes in the average income of the age subgroups fostered an increase in the level of inequality. The changes observed in the structure of the household population with respect to the education of the household head adversely affected the level of income inequality, contributing to its increase and adding to the within-group and between-group component alike. However, relative changes in the average income of the subgroups distinguished by education supported a decrease in the level of inequality.

## 5. Discussion and Conclusions

The empirical aim of the study was to assess the extent to which education and age affect household income inequality, as well as whether their impact is stable over time. Therefore, in order to attain the aim, our analysis was based on micro-data obtained from the EU-SILC database and employed MLD as a research tool.

The literature on the subject indicates education as one of the most crucial factors influencing income inequality. However, the impact of education on income inequality is yet to be fully understood and may differ from economy to economy. The association of education and income inequality is also frequently examined in empirical studies, and their results—sometimes contradictory—reflect the complexity of the discussed issue. Chevan and Stokes [53] even refer to education as “the Pandora’s box of income inequality”, claiming that both low and high levels of education can foster income inequality. Checchi [54] claims that facilitated access to tertiary education can increase earning opportunities of the poorest groups of the population and can lead to a reduction of income inequality. Moreover, Rodríguez-Pose and Tselios [55] confirm that education is considered one of the most powerful known instruments for reducing income inequality. However, they emphasize that an increase in the share of the population with tertiary education leads to a reduction of the value of education and, in the longer term, to a decrease in the wages of some workers with tertiary education.

The current study confirmed an association between the level of education and the average income of the groups distinguished on this basis. Based on the obtained results, it can be concluded that the education level of the household head remains an important determinant of household income inequality in Poland, despite the decline in the “educational bonus”. Although the predominance of average income of households with “high” education of the household head, over the national average, has almost halved (from 77% to 40%), the differences in the level of education still account for 16% of income inequality. The current results are in line with the results of other empirical studies on the causes of income inequality [32,52,56]. All of these studies confirmed that, in the post-transition countries, the level of education remains one of the important sources of income inequality.

The current study also found that differences in the age of the household head had a smaller effect on income inequality than the level of education. Since the results have shown that only 2.6% of income inequality can be explained by the age of the household head, the contribution of this characteristic to total income inequality may seem to be negligible. However, the results prove that the aging of the population can foster income inequality in Poland and may increase the impact of age on income inequality. The results achieved are in line with the report of the RAND Corporation [57], which points out that income inequality in Europe is sensitive to an aging of population.

Brandolini and D’Alessio [58] indicate that the age structure of population can affect income inequality because the amount and composition of personal incomes (from work, property, and transfer) vary over life, and also because individual experiences mirror the different historical periods. Typically, an individual’s income over the entire life is hump-shaped: it usually increases from the moment of entering the labor market to approximately the age of 65 and then decreases when the income from work is replaced by the income from pension. However, a precise form of the hump-shaped curve is not fixed and responds to redistribution between generations due to changes in labor market relations and the trends in economic and social policies. The current study does not reveal the inverted U-shape (hump-shaped) pattern in household incomes in Poland, but it does confirm the deterioration of the relative income situation in the case of households headed by the oldest persons.

Household income may not confirm the hump-shaped pattern due to the fact that the household, by accumulating and redistributing the income of its members, acts as a redistributor of resources [59]. Households combine the incomes of all their members, thereby equalizing their level and eliminating the income disparities within the household, and this, in turn, reduces the income inequality between households.

However, the aging of the population may substantially reduce the redistributive role of the household. Therefore, it can be concluded that the higher share of older people may contribute to an increase in income inequality between groups, as the income from pension in Poland is more homogeneous than the income from work in younger groups.

In addition to empirical research, the current paper sought to situate Theil’s approach in the context of scholarly writings since 1967, and the Mean Logarithmic Deviation was selected because of its attractive properties. Firstly, the choice of the MLD resulted from an emphasis on the sensitivity of this measure to the lower tail of the income distribution. Secondly, as discussed at length in this paper, the MLD shares many of the well-established properties of the Generalized Entropy measures, the median-normalized inequality measures, and path-independent inequality measures. The MLD also respects the principle of monotonicity in distance and is decomposable for arbitrary partitions with the path-independence property. As Cowell and Flachaire [37] showed, the lack of the principle of monotonicity in distance may have strong implications in empirical studies. Moreover, the properties of additive decomposition fulfilled by MLD cannot be overestimated, namely the between-group and within-group components are independent, and the weights of the within-group expressions do not depend on the subgroup means.

The empirical study was based on the income data derived directly from the household survey. When using such data, it should be remembered that low- and high-income households can be underrepresented in the survey data due to the fact that people often refuse to provide any information about their income or understate it. As a result, the measures of income inequality can be underestimated. Furthermore, different approaches to selecting the head of the household can affect the results of empirical studies.

## Figures and Tables

**Table 1:** Summary statistics for the subgroups were distinguished based on the age of the household head.

Age	Population Share		Income Share		Relative Mean		*MLD*	
	2005	2019	2005	2019	2005	2019	2005	2019
18–35	0.170	0.121	0.177	0.138	1.038	1.142	0.276	0.158
36–49	0.378	0.345	0.361	0.364	0.957	1.054	0.257	0.156
50–64	0.369	0.424	0.378	0.407	1.025	0.960	0.215	0.138
above 65	0.083	0.109	0.084	0.090	1.004	0.825	0.097	0.103

Source: Own computations based on the EU-SILC data.

**Table 2:** Results of the *MLD* static decomposition.

	2005			2019		
	*MLD_W_*	*MLD_B_*	Ratio	*MLD_W_*	*MLD_B_*	Ratio
Age	0.2313	0.0006	0.3%	0.1427	0.0039	2.6%
Education	0.1953	0.0365	15.8%	0.1231	0.0234	16.0%

Source: Own computations based on the EU-SILC data.

**Table 3:** Summary statistics for the subgroups distinguished by the education of the household head.

Education	Population Share		Income Share		Relative Mean		*MLD*	
	2005	2019	2005	2019	2005	2019	2005	2019
Low	0.215	0.110	0.156	0.077	0.725	0.701	0.162	0.104
Medium	0.659	0.660	0.620	0.601	0.941	0.911	0.204	0.116
High	0.127	0.230	0.224	0.322	1.773	1.398	0.206	0.153

Source: Own computations based on the EU-SILC data.

**Table 4:** Results of the *MLD* dynamic decomposition.

	Within-Group Component		Between-Group Component	
	Inequality Effect	Allocation Effect		Income Effect
Age	−98.1%	−5.8%	0.0%	3.9%
Education	−92.4%	5.8%	9.9%	−23.2%

Source: Own computations based on the EU-SILC data.

## Data Availability

Restrictions apply to the availability of these data. Data were obtained from Eurostat and are only available from Eurostat.
